# Protective effects of curcumin/cyclodextrin polymer inclusion complex against hydrogen peroxide‐induced LO2 cells damage

**DOI:** 10.1002/fsn3.2787

**Published:** 2022-02-24

**Authors:** Jianping Chen, Jiarui Li, Tugui Fan, Saiyi Zhong, Xiaoming Qin, Rui Li, Jialong Gao, Yuanwei Liang

**Affiliations:** ^1^ 74780 College of Food Science and Technology Guangdong Provincial Key Laboratory of Aquatic Product Processing and Safety Guangdong Provincial Engineering Technology Research Center of Seafood Guangdong Province Engineering Laboratory for Marine Biological Products Key Laboratory of Advanced Processing of Aquatic Product of Guangdong Higher Education Institution Guangdong Ocean University Zhanjiang China; ^2^ Collaborative Innovation Center of Seafood Deep Processing Dalian Polytechnic University Dalian China; ^3^ 74780 College of Chemistry and Environment Guangdong Ocean University Zhanjiang China

**Keywords:** curcumin, cyclodextrin polymer, hydrogen peroxide, liver injury, protective effects

## Abstract

The objective of the present study was to explore the protective effects of the curcumin/cyclodextrin polymer (CUR/CDP) inclusion complex on hydrogen peroxide (H_2_O_2_)‐induced LO2 cells damage. In this study, a H_2_O_2_‐induced cells oxidative injury model was established to test the protective effects of the CUR/CDP inclusion complex. The cell viability of cells was detected by the thiazolyl blue tetrazolium bromide (MTT) assay. The extracellular lactate dehydrogenase (LDH) activity, catalase (CAT) activity, and malondialdehyde (MDA) level were detected by assay kits. The cellular reactive oxygen species (ROS) level was detected using the dichlorodihydrofluorescein (DCF) fluorescence assay. Western blotting analysis was conducted to assess the changes of phosphorylated‐p53 and caspase‐3. The results showed that 700 μM H_2_O_2_‐treated LO2 cells for 3 h resulted in a significant decrease of cell viability to 53.00 ± 1.68%, which established the cell oxidative injury model. Cells treated with H_2_O_2_ led to a significant increase of extracellular LDH activity, MDA content, and ROS level, and decreased CAT activity. Treatment with CUR/CDP significantly reversed the changes of the above indicators. Moreover, CUR/CDP treatment at 20 and 40 μg/ml inhibited H_2_O_2_‐induced increase in phosphorylated‐p53 and caspase‐3 expression, indicating that CUR/CDP suppressed cell apoptosis to alleviate liver injury. The results of those studies demonstrated that CUR/CDP had a protective effect on the oxidative damage of LO2 cells, and it could be developed as a new type of natural liver protection product to apply in the prevention of liver injury.

## Graphical abstract

We investigated the protective effect of the curcumin/cyclodextrin polymer (CUR/CDP) inclusion complex on hydrogen peroxide (H_2_O_2_)‐induced LO2 cells damage. CUR/CDP was found to possess protective effects against the H_2_O_2_‐induced liver injury. Further mechanistic theory showed that the CUR/CDP treatment suppressed cell apoptosis to alleviate liver injury through downregulation of phosphorylated‐p53 and caspase‐3 levels. This research elucidates the protective mechanism of CUR/CDP against hydrogen peroxide‐induced liver injury. This novel complex of curcumin has the potential to prevent hydrogen peroxide‐induced liver injury.

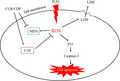

## 1 INTRODUCTION

It is well known that as one of the most important organs in human body, liver is involved in drug metabolism and detoxification (Mariee et al., 2012; Nagata et al., 2007; Wang et al., 2012). Liver injury is considered to be the result of these metabolic reactions. Numerous studies have also shown that oxidative stress has been associated with the pathogenesis of various kinds of liver damage (Li et al., 2016). Oxidative stress results from a disturbance in the balance between prooxidants and antioxidants. Living organisms have multiple antioxidant systems, including antioxidant enzymes, such as superoxide dismutase (SOD), glutathione peroxidase (GPx), and catalase (CAT), and nonenzymatic antioxidants, such as vitamins C, glutathione (GSH), and so on. When a liver suffers from heavy metals, xenobiotics, and drugs, the reactive oxygen species (ROS) level will show a sharp increase in a short time, whereas endogenous antioxidants such as SOD and CAT are exhausted (Zhao et al., 2016). Thus, excessive ROS such as hydrogen peroxide (H_2_O_2_) in hepatocytes could induce oxidative stress, which results in cell injury and cell apoptosis (Kwok et al., 2010). Therefore, protection of hepatocytes from oxidative injury and cell death should be an effective strategy for preventing liver diseases.

Plant phytochemicals are found to be effective hepatoprotective agents, which can prevent all kinds of liver diseases (Cui et al., 2014). These phytochemicals, including flavonoids (Liu et al., 2014), polysaccharides (Zhang et al., 2017), and peptides (Ma et al., 2021), play a crucial role in protecting liver from ROS‐mediated damage. Curcumin (CUR) extracted from dry rhizome of *Curcuma longa* is a diketone compound and has been generally applied in food and Asian medicine (Lu et al., 2015). Several studies have also found that CUR attenuated oxidative stress and possessed hepatoprotective actions on liver injury due to its strong antioxidant activity (Ramsés & José, 2014). However, the poor solubility of CUR results in its low bioavailability, which limits its clinical application. For this reason, in our previous study, curcumin/β‐cyclodextrin polymer (CUR/CDP) inclusion complex was prepared using β‐cyclodextrin polymer (CDP) as a host molecule to improve curcumin's solubility (Chen et al., 2018). Yet, little information has been reported about the detailed effects of CUR/CDP on oxidative injury.

As an important component of ROS, H_2_O_2_ is generated under various physiological or pathological conditions and leads to oxidative injury (Li et al., 2012). H_2_O_2_ is a common inducer to induce oxidative stress in vitro models of oxidative damage (Chen et al., 2021; Zhao et al., 2021). Besides, in the study of liver injury, LO2 cell line has been commonly used (Hu et al., 2015; Wang et al., 2008). Therefore, in this work, a model of H_2_O_2_‐induced LO2 cell oxidative damage was established and the protective effect of CUR/CDP against oxidative stress induced by H_2_O_2_ in LO2 cells was explored.

## 2 MATERIALS AND METHODS

### 2.1 Materials and reagents

Three percent (w/v) H_2_O_2_ was obtained from Shandong LIRCON Medical Technology Incorporated Company (Dezhou, Shandong, China). Curcumin and thiazolyl blue tetrazolium bromide (MTT) were supplied from Sigma Company (St. Louis, MO, USA). Bicinchoninic acid (BCA) kits and 6‐carboxy‐2′‐7′‐dichlorofluorescein diacetate (DCFH‐DA) were provided by Beyotime Biotechnology (Shanghai, China). Lactate dehydrogenase (LDH), malondialdehyde (MDA), and CAT assay kits were purchased from Nanjing Jiancheng Biology Engineering Institute (Nanjing, Jiangsu, China). Fetal bovine serum (FBS), Dulbecco's Modified Eagle's Medium (DMEM), and trypsin were purchased from Gibco (Grand Island, NY, USA). Cyclodextrin polymer (CDP) was prepared in our laboratory according to the method reported by Zhang et al. (Zhang et al., 2011). The experimental antibodies were provided by Cell Signaling Technology Inc. (Beverly, Massachusetts, USA). The experimental LO2 cells were provided by Shanghai Fuheng Biotechnology Co., Ltd (Shanghai, China).

### 2.2 Preparation of the CUR/CDP inclusion complex

The CUR/CDP inclusion complex was prepared according to the method reported by Chen et al. (Chen et al., 2018). In brief, 4 g CDP and 1 g curcumin were weighed and added to a mortar for grinding uniformly, then poured into a conical flask with 50 ml of distilled water. After 5 min of ultrasonication, the mixed solution was placed on a magnetic stirrer for 48 h at room temperature (RT). Then, undissolved curcumin in the solution was removed by filtration. Finally, CUR/CDP inclusion complex was obtained after the solution was concentrated and dried for 48 h in a vacuum oven at 60°C.

### 2.3 Cell culture and drug treatment

LO2 cells were cultured in DMEM containing 10% FBS, 50 units/ml streptomycin, and 100 units/ml penicillin. The cells were grown at 37°C under a humidified (5% CO_2_, 95% air) atmosphere.

To establish a cell model of oxidative injury, LO2 cells (8 × 10^4^ cells/well) were planted in 96‐well plates and incubated for 24 h. Then, 100 μL of 400, 500, 600, 700, 800, and 900 µM H_2_O_2_ was added, followed by cultivation for 3, 6, and 12 h, respectively.

To estimate the effects of CUR/CDP on LO2 cells, cells (8 × 10^4^ cells/well) were pretreated with CUR/CDP at indicated concentration for 12 h before exposure of H_2_O_2_. Then, 700 µM H_2_O_2_ was added followed by incubation at 37°C for 3 h. In the H_2_O_2_‐treated group, the cells underwent the same procedure without CUR/CDP pretreatment. And in the control group, the cells were treated in the same procedure without both CUR/CDP and H_2_O_2_ treatments. After the cells were incubated at 37°C for 3 h, the following tests were carried out.

### 2.4 Measurement of cell viability

LO2 cells with a density of 8 × 10^4^ cells per well were treated with different group treatments at indicated concentrations for the indicated times. Then, the MTT assay was used to measure cell viability, i.e., after treatment of the cells, 20 µL MTT solution (5 mg/ml) was added to each culture well followed by incubation for 4 hr. Following medium removal, 150 µL of dimethyl sulfoxide (DMSO) was added to solubilize the formazan crystal. Finally, the absorbance of the formazan solution was assessed using a microplate reader (Varioskan Flash, Thermo, USA) at 570 nm.

### 2.5 Measurement of extracellular LDH activity

LO2 cells (1 × 10^6^ cells per well) were first plated in the 6‐well plate for 24 h. CUR/CDP (10, 20, and 40 µg/mL) was subsequently added to the wells followed by pretreatment for 12 h before exposure of H_2_O_2_. After that, the LDH activity in the culture medium was detected using a LDH assay kit according to manufacturer's protocols. Absorbance was read on the microplate photometer (Varioskan Flash, Thermo, USA) at 450 nm.

### 2.6 Estimation of the intracellular ROS level

The relative level of intracellular ROS was detected using the 2′,7′ dichlorofluorescein (DCF) fluorescence assay. Briefly, the cells were seeded at a density of 8 × 10^4^ cells per well in 96‐well plates and exposed to different concentrations of CUR/CDP for 12 h followed by H_2_O_2_ (700 µM) treatment for 3 h. Following the treatment, the cells were cultured for 20 min at 37°C in the serum‐free medium supplemented with 10 µM of DCFH‐DA. Then, extracellular dye was removed by washing the cells three times with PBS. The fluorescence intensity was immediately measured with a microplate photometer (Varioskan Flash, Thermo, USA) at 488 nm excitation and 525 nm emission, respectively.

### 2.7 Measurement of CAT activity

LO2 cells were pretreated with or without CUR/CDP (10–40 µg/ml) for 12 h and treated with or without H_2_O_2_ (700 µM) for additional 3 h. After that, cells were resuspended in lysis buffer. After centrifugation at 12,000 × g for 5 min at 4°C, the supernatant obtained from lysates was used for the following experiments. The CAT activity was measured by the commercial assay kit, according to manufacturer's protocols (Nanjing Jiancheng Co., China).

### 2.8 Measurement of the MDA level

LO2 cells were seeded 1 × 10^6^ cells per well in 6‐well plates and pretreated with or without CUR/CDP at 10–40 µg/ml for 12 h and then challenged with or without 700 µM H_2_O_2_ for additional 3 h. Then, after aspiration of the medium, extraction solution was added to lyse the cells. The supernatant obtained from lysates was used for the following experiments. The level of MDA was determined using the commercially available assay kit, according to manufacturer's instructions (Nanjing Jiancheng Co., China).

### 2.9 Western blotting assay

Treated LO2 cells were obtained and the whole cellular protein was extracted in lysis buffer. Then, protein concentrations were determined using the BCA assay kit (Beyotime Biotechnology, Shanghai, China). The equal amounts of protein loaded per lane were separated on 8% sodium dodecyl sulfate –polyacrylamide gel (SDS–PAGE). After that, proteins were electrotransferred to a nitrocellulose membrane. The membrane was cultured with primary antibodies against phosphorylated‐p53, caspase‐3, or β‐actin at 1:1000 dilution overnight, then processed with secondary antibodies conjugated with horseradish peroxidase at 1:2000 dilution for 2 h. Protein bands were observed using the enhanced chemiluminescence reagent (ECL).

### 2.10 Statistical analysis

All experiments were done at least three times and all data were presented as mean ± standard deviation (S.D.). SPSS 22.0 software (IBM Corporation, Armonk, NY, USA) was utilized for statistical analysis. The comparison between two groups was analyzed by two‐tailed Student's *t*‐test.

## 3 RESULTS AND DISCUSSION

### 3.1 Establishment of the cell oxidative injury model

**FIGURE 1:**
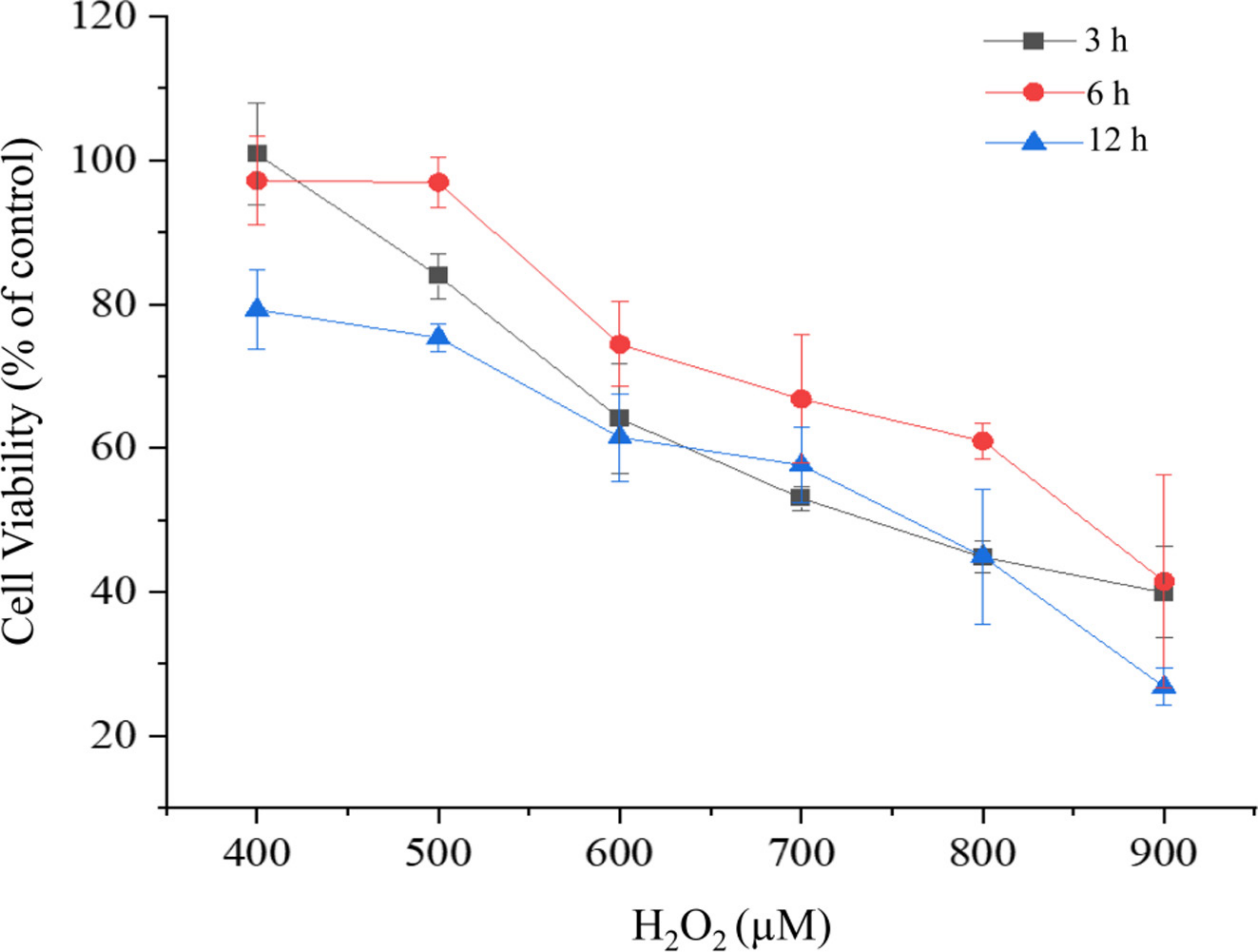
Effects of H_2_O_2_ solution at different concentrations and treating times on LO2 cell viability. Cytotoxic effect of H_2_O_2_ on LO2 cells was measured by the thiazolyl blue tetrazolium bromide (MTT) assay. LO2 cells were treated with H_2_O_2_ (400–900 µM) for 3, 6, and 12 h, respectively

First, to establish a model of the oxidative injury, MTT assay was utilized for measuring the effects of H_2_O_2_ at 400–900 µM on cell viability of LO2 cells. MTT assay which was first introduced by Mosmann has become the most common method to measure cell viability (Nga et al., 2020). It is based on the mitochondrial succinate dehydrogenases in viable cells, which reduces MTT into water‐insoluble purple formazan crystals (Boncler et al., 2014). Briefly, human normal liver cells LO2 were exposed to H_2_O_2_ at various concentrations (400, 500, 600, 700, 800, and 900 µM) for 3, 6, and 12 h, respectively. The results are shown in Figure 1. With the H_2_O_2_ concentration and treating time increased, we found that H_2_O_2_ significantly inhibited LO2 cells viability in a concentration‐dependent manner but not time‐dependent manner. For instance, after cells were treated with H_2_O_2_ (700 µM), the cell viability was decreased to 53.00 ± 1.68% (3 h), 66.80 ± 8.89% (6 h), and 57.63 ± 5.17% (12 h), respectively. However, cells exposed to H_2_O_2_ (900 µM) exhibited reduced cell viability from 53.00 ± 1.68% (3 h), 66.80 ± 8.89% (6 h), 57.63 ± 5.17% (12 h) to 39.90 ± 6.35% (3 h), 41.48 ± 14.80% (6 h), 26.78 ± 2.55% (12 h), respectively. These results suggested that H_2_O_2_ could induce LO2 cells injury. In general, to establish the oxidative injury model, the cell survival rate usually decreases by 30%–50% (Zhao et al., 2016). According to the above results, we selected 700 µM H_2_O_2_ for 3 h as the optimal condition to treat cells in all subsequent experiments.

### 3.2 Effect of CUR/CDP on H_2_O_2_ induced the loss of cell viability

**FIGURE 2:**
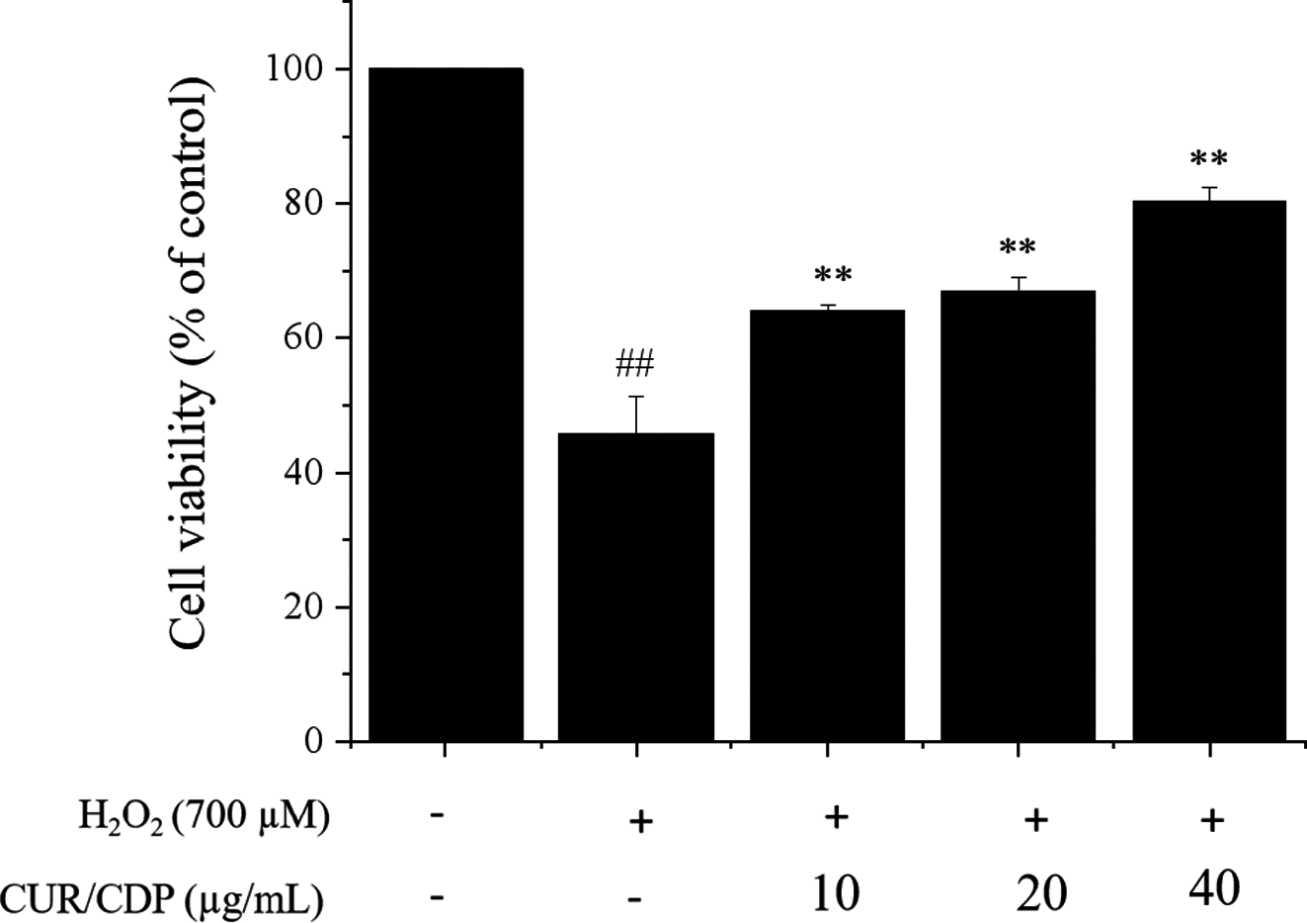
Protective effects of curcumin/cyclodextrin polymer (CUR/CDP) on H_2_O_2_‐treated LO2 cells. LO2 cells were preincubated with CUR/CDP (10–40 µg/ml) for 12 h and then stimulated with H_2_O_2_ (700 µM) for additional 3 h. Cell viability was determined using the thiazolyl blue tetrazolium bromide (MTT) assay. *p* < 0.01 (**) means that columns between H_2_O_2_ group and CUR/CDP groups are significantly different. *p* < 0.01 (##) means that columns between control group and H_2_O_2_ group are significantly different

In order to investigate if CUR/CDP enhanced the cell viability of H_2_O_2_‐injured LO2 cells, MTT assay was used to detect cell viability. Briefly, LO2 cells were pretreated with CUR/CDP (10–40 µg/mL) for 12 h and co‐incubated with 700 µM H_2_O_2_ for another 3 h. The results are exhibited in Figure 2. After exposure of LO2 cells to 700 µM H_2_O_2_ for 3 h, cell viability was significantly decreased to 45.87 ± 5.46%. However, pretreatment of LO2 cells with CUR/CDP at diverse concentrations (10–40 µg/ml) for 12 h markedly increased the cell viability in a dose‐dependent manner. For example, compared to the model group, CUR/CDP at 40 µg/ml markedly increased from 45.87 ± 5.46% to 80.36 ± 1.83%. These results indicated that CUR/CDP could reverse the H_2_O_2_‐induced cell death.

### 3.3 Effect of CUR/CDP on LDH release in H_2_O_2_‐injured LO2 cells

**FIGURE 3:**
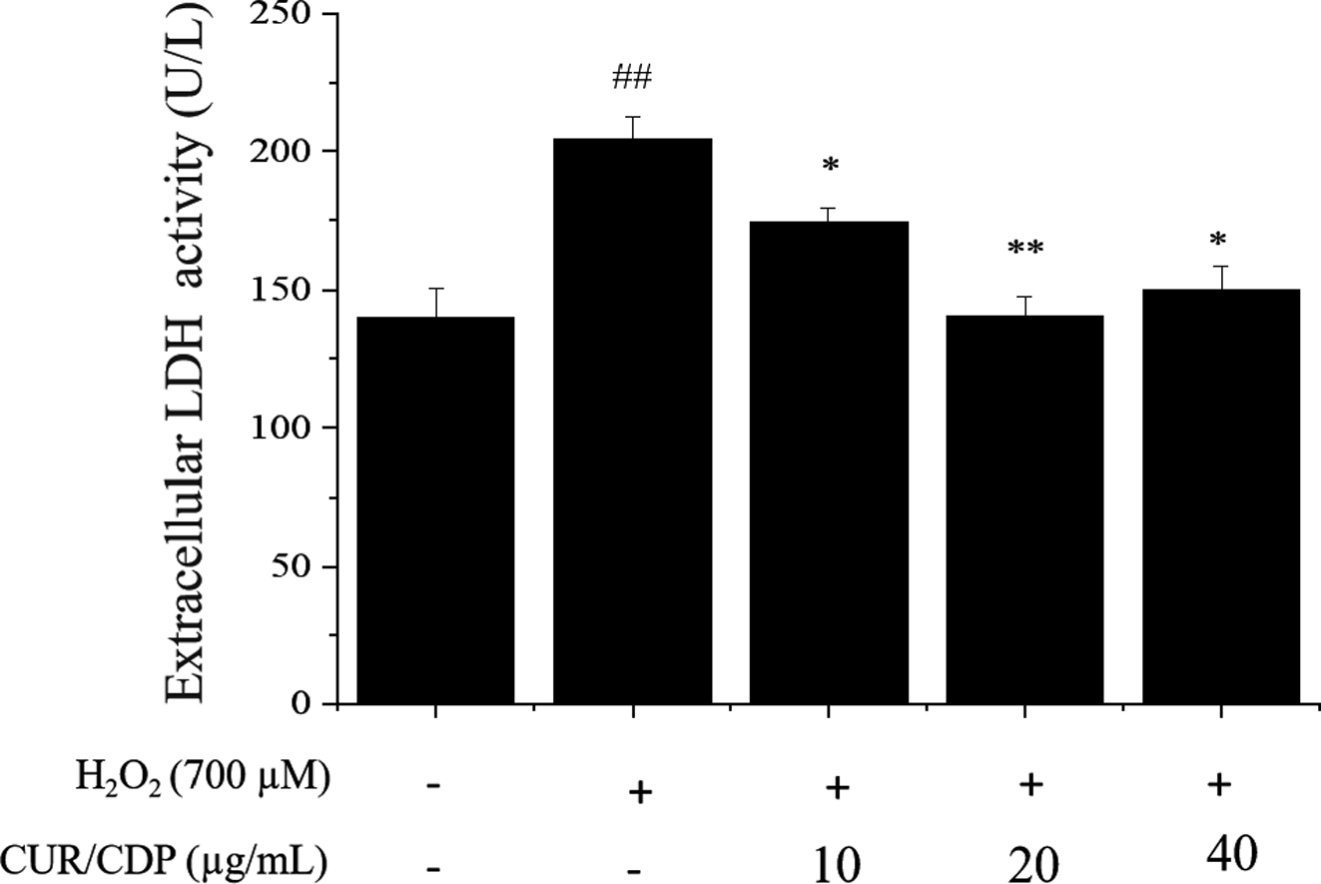
Effects of curcumin/cyclodextrin polymer (CUR/CDP) on lactate dehydrogenase (LDH) leakage in H_2_O_2_‐treated LO2 cells. LO2 cells were preincubated with CUR/CDP (10–40 µg/ml) for 12 h and then stimulated with H_2_O_2_ (700 µM) for additional 3 h. The LDH assay kit was used to determine the release of LDH. *p* < 0.01 (**) or *p* < 0.05 (*) means that columns between H_2_O_2_ group and CUR/CDP groups are significantly different. *p* < 0.01 (##) means that columns between control group and H_2_O_2_ group are significantly different

It is well known that LDH is an indicator of cellular damage (Bagchi et al., 1995). Thus, the LDH kit assay was used to determine the LDH activity in the culture medium. The cultured LO2 cells were incubated in the presence of 10, 20, or 40 µg/ml CUR/CDP for 12 h and the activity of extracellular LDH was measured after 3 h of H_2_O_2_ incubation as an index of cytotoxicity. The results are shown in Figure 3. A significant increase in LDH release was observed in the H_2_O_2_ treatment group. The activity of extracellular LDH in the H_2_O_2_ treatment group increased from 140.24 ± 10.42% (control) to 204.4 ± 8.01%, compared with the control group. It was speculated that cell membrane damage caused by hydrogen peroxide resulted in the release of intracellular LDH into extracellular LDH, which led to the enhancement of extracellular LDH activity (Wang et al., 2018). Pretreatment with different concentrations of CUR/CDP decreased the extracellular LDH activity, but dose‐dependent manner was not shown. For instance, CUR/CDP at 20 µg/ml and 40 µg/ml markedly reduced the activity of extracellular LDH to 140.62 ± 7.11% and 150.19 ± 8.62%, respectively. These results indicated that CUR/CDP could suppress LDH leakage by inhibiting cell membrane damage in H_2_O_2_‐injured LO2 cells.

### 3.4 CUR/CDP inhibits induction of ROS generation in H_2_O_2_‐injured LO2 cells

**FIGURE 4:**
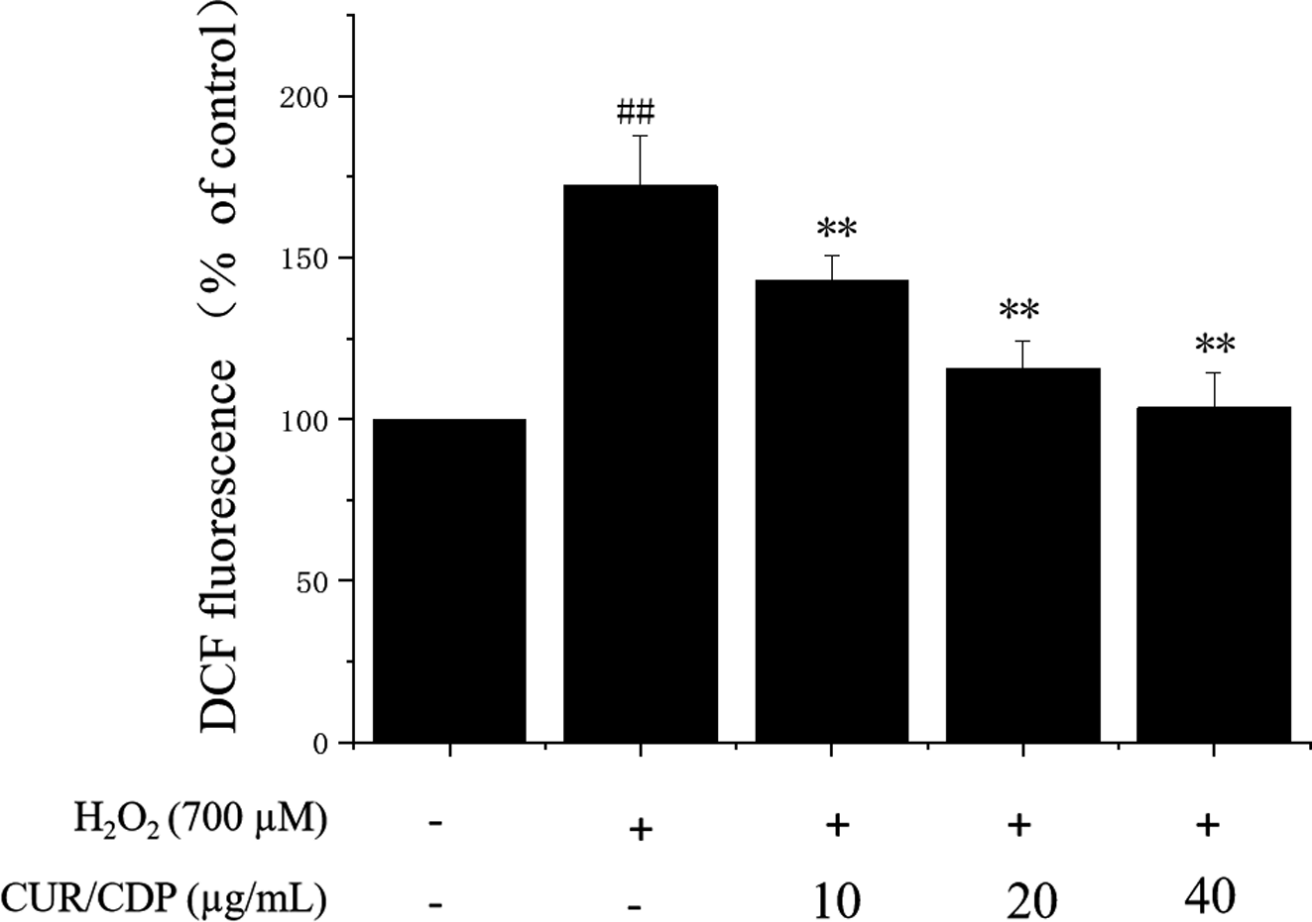
Effect of curcumin/cyclodextrin polymer (CUR/CDP) pretreatment on intracellular reactive oxygen species (ROS) in H_2_O_2_‐induced LO2 cells damage. LO2 cells were preincubated with CUR/CDP (10–40 µg/ml) for 12 h and then stimulated with H_2_O_2_ (700 µM) for additional 3 hr. 2′,7′ dichlorofluorescein (DCF) fluorescent density was detected by the ROS assay kit. *p* < 0.01 (**) means that columns between H_2_O_2_ group and CUR/CDP groups are significantly different. *p* < 0.01 (##) means that columns between control group and H_2_O_2_ group are significantly different

Reactive oxygen species are a kind of free radical active substances produced during normal physiological events. However, research showed that production of excess ROS damaged cellular DNA, lipids, and proteins, leading to cell death (Singh et al., 2011). Liu and coworkers also demonstrated that ROS played a critical role in H_2_O_2_‐dependent cell death (Liu et al., 2010). To explore the effect of CUR/CDP on H_2_O_2_‐induced oxidative stress, ROS generation in LO2 cells was detected by measuring the fluorescent dichlorofluorescein (DCF). This assay is based on nonfluorescent probe DCFH‐DA oxidizing to fluorescent product DCF in the presence of ROS (Zou et al., 2017). The results shown in Figure 4 demonstrated that an increase in the intensity of DCF was observed in the cells exposed to 700 µM H_2_O_2_ for 3 h, compared with the control group. However, the exposure of LO2 cells to CUR/CDP dose‐dependently decreased fluorescent intensity of DCF, indicating that CUR/CDP inhibited H_2_O_2_‐induced ROS generation.

### 3.5 Effects of CUR/CDP on CAT activity and MDA content in H_2_O_2_‐injured LO2 cells

**FIGURE 5:**
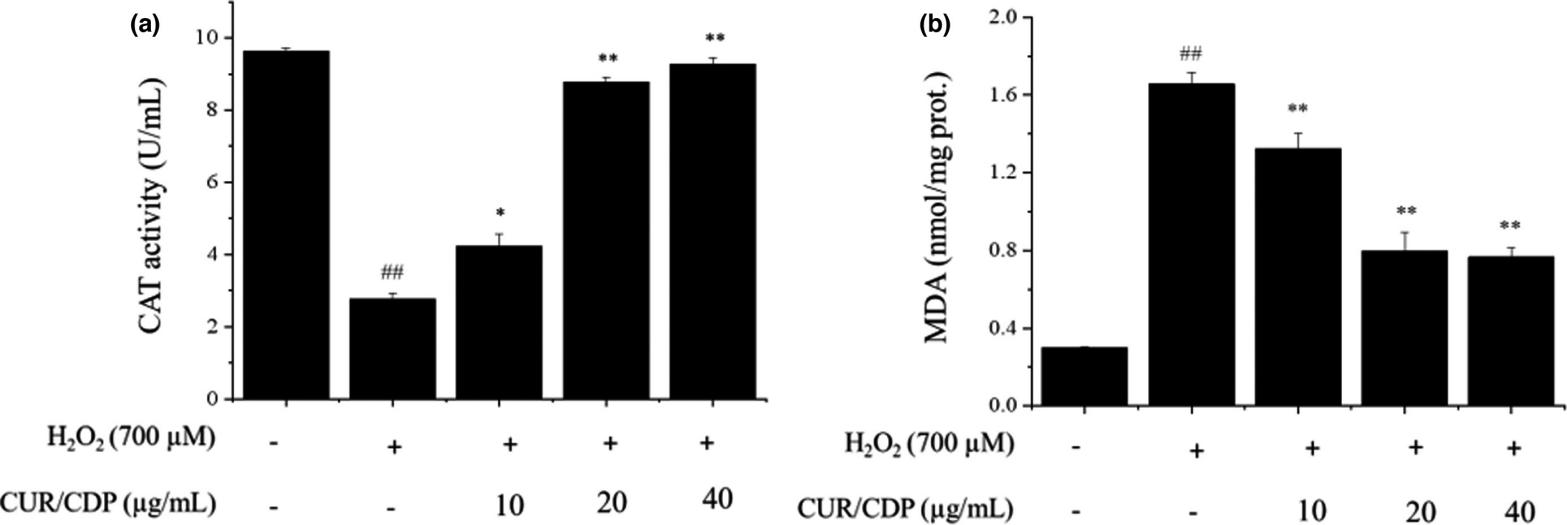
Effect of curcumin/cyclodextrin polymer (CUR/CDP) pretreatment on the activity of catalase (CAT) (a) and malondialdehyde (MDA) content (b) in LO2 cells treated with H_2_O_2_. LO2 cells were preincubated with CUR/CDP (10–40 µg/ml) for 12 h and then stimulated with H_2_O_2_ (700 µM) for additional 3 h. The intracellular MDA content and CAT activity were detected with spectrophotometry. *p* < 0.01 (**) or *p* < 0.05(*) means that columns between H_2_O_2_ group and CUR/CDP groups are significantly different. *p* < 0.01 (##) means that columns between control group and H_2_O_2_ group are significantly different

In cellular antioxidant enzymes, CAT is identified as the most effective enzyme associated with the detoxification of H_2_O_2_ and can prevent cell damage for scavenging ROS (De Bleser et al., 1999; Xiao et al., 2008). Besides, research reported that liver damage was associated with heightened lipid peroxidation and generation of lipid radicals (Lu & Cederbaum, 2008; Tsukamoto & Lu, 2001). MDA, an end product of lipid peroxidation, reflects oxidative damage of cell membrane and the extent of lipid peroxidation. To investigate whether CUR/CDP had shown antioxidative effect on H_2_O_2_‐injured LO2 cells, CAT activity and MDA content were measured by assay kits. The results shown in Figure 5 demonstrated that after exposure of LO2 cells to 700 µM H_2_O_2_ for 3 h, compared to control cells, the activity of CAT was decreased from 9.63 ± 0.09 U/ml (control group) to 2.78 ± 0.16 U/ml (Figure 5a), and MDA levels were increased from 0.30 ± 0.00 nmol/mg protein (control group) to 1.66 ± 0.06 nmol/mg protein (Figure 5b), indicating that H_2_O_2_ inhibited CAT activity and promoted production of MDA. However, CUR/CDP (10–40 µg/ml) pretreatment dose‐dependently reversed a decrease in the activity of CAT and lowered MDA content. These results showed that CUR/CDP was capable of antioxidant action.

### 3.6 Effects of CUR/CDP on phosphorylated‐p53 and caspase‐3 expression in H_2_O_2_‐injured LO2 cells

**FIGURE 6:**
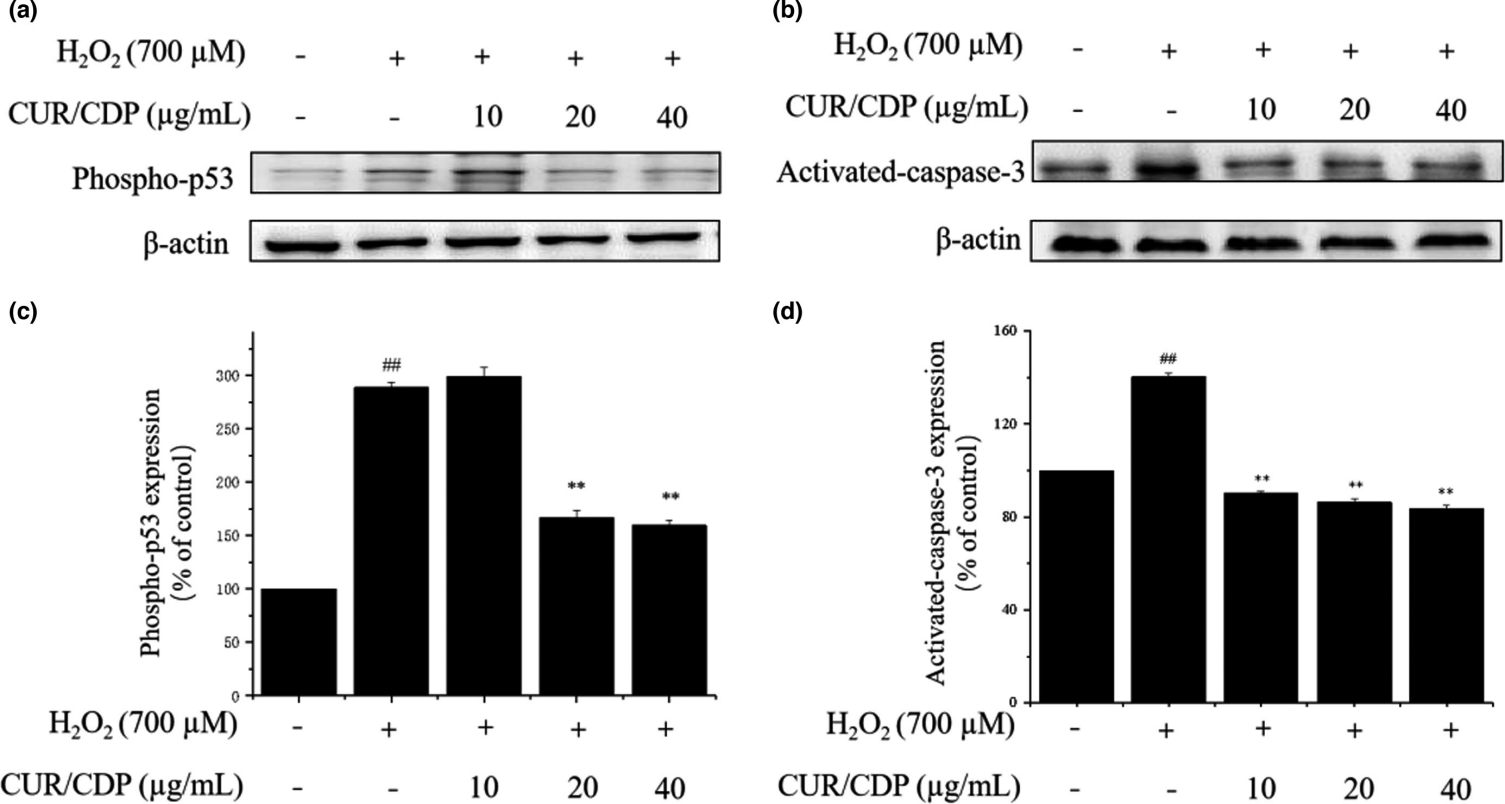
Effects of curcumin/cyclodextrin polymer (CUR/CDP) on the expression levels of phosphorylated‐p53 and caspase‐3. The expression levels of phosphorylated‐p53 (a) and caspase‐3 (b) were detected by Western blotting analysis. LO2 cells were preincubated with CUR/CDP (10–40 µg/ml) for 12 h and then stimulated with H_2_O_2_ (700 µM) for additional 3 h. Protein expression level of phosphorylated‐p53 (c) and caspase‐3 (d) as percentage of control. *p* < 0.01 (**) means that columns between H_2_O_2_ group and CUR/CDP groups are significantly different. *p* < 0.01 (##) means that columns between control group and H_2_O_2_ group are significantly different

It is clear that oxidative stress can induce cell apoptosis (Wu et al., 2018). The nuclear transcription factor p53 plays a vital role in cellular responses to oxidative stresses (Liu & Xu, 2011). p53 can increase cellular oxidative stresses by inhibiting the expression of antioxidant genes, thereby inducing cell apoptosis. Phosphorylation of p53 is closely associated with p53 activation during cellular stress response (Long et al., 2007). Caspases, a family of cysteine acid proteases, play a critical role in mediating cell apoptosis, and caspase‐3 is the primary executioner of apoptosis in mammalian cells (Chen et al., 2020). p53 activation causes mitochondrial dysfunction, which leads to the activation of caspase‐3 by releasing apoptotic factors into cytoplasm (Chen et al., 2014). Therefore, in order to verify whether p53 was connected with the process of oxidative stress‐induced cell apoptosis, we investigated phosphorylated‐p53 and its downstream protein caspase‐3 levels using Western blotting assay. The results shown in Figure 6 demonstrated that H_2_O_2_ alone treatment resulted in a significant enhancement in the expression levels of phosphorylated‐p53 (Figure 6a) and activated‐caspase‐3 (Figure 6b), compared with the control group. Exposure of LO2 cells to CUR/CDP at 20 and 40 µg/ml markedly downregulated phosphorylated‐p53 and activated‐caspase‐3 levels. For example, treatment of H_2_O_2_ increased phosphorylated‐p53 and activated‐caspase‐3 expression in LO2 cells to 288.61 ± 4.50% and 140.41 ± 1.51%, respectively (Figure 6c and d). Pretreatment of LO2 cells with CUR/CDP at 20 and 40 µg/ml prior to H_2_O_2_ decreased phosphorylated‐p53 and activated‐caspase‐3 expression to 167.09 ± 6.86%, 86.34 ± 1.40% and 159.49 ± 4.79%, 83.72 ± 1.32%, respectively (Figure 6c and d). The above results indicated that CUR/CDP could inhibit phosphorylated‐p53 and activated‐caspase‐3 expression induced by H_2_O_2_ in LO2 cells.

**FIGURE 7:**
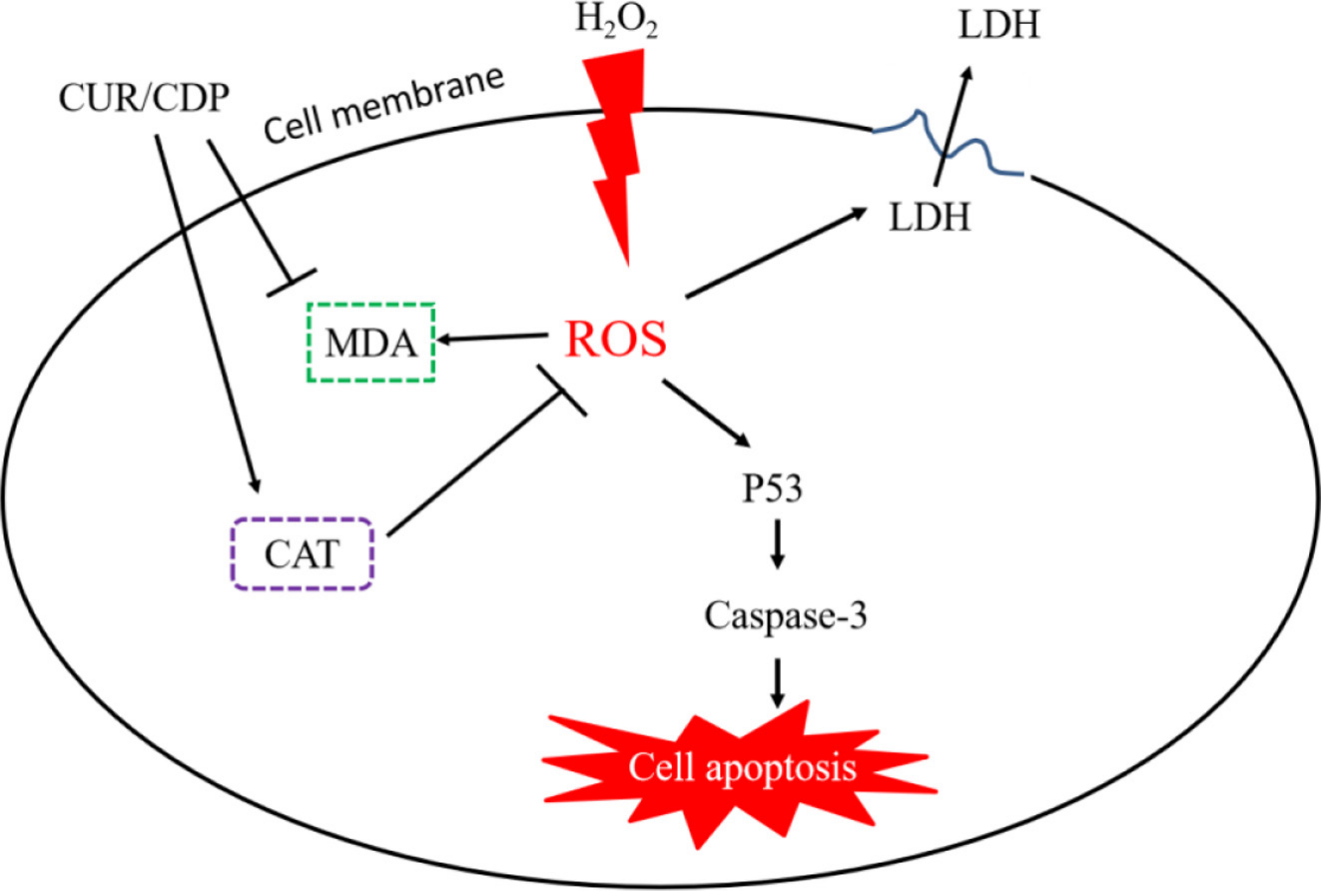
Schematic diagram of the possible mechanisms of curcumin/cyclodextrin polymer (CUR/CDP) that protects against H_2_O_2_‐induced cell death in LO2 cells

As mentioned above, the proposed mechanisms for the hepatoprotective effect of CUR/CDP on liver injury induced by H_2_O_2_ are shown in Figure 7. First, H_2_O_2_ treatment enhanced the intracellular ROS level. Overproduction of ROS resulted in the CAT activity decrease, MDA content increase, LDH leakage and induced cell apoptosis. However, pretreatment with CUR/CDP could reverse the changes in these indicators, which were mainly attributed to its antioxidant capacity resulting in the reduction of oxidative stress, thereby downregulating phosphorylated‐p53 and caspase‐3 expression to inhibit cell apoptosis.

## 4 CONCLUSIONS

To sum up, our study indicates that CUR/CDP exerts protective effects against H_2_O_2_‐induced liver injury. These beneficial effects may be associated with the antioxidant property of CUR/CDP, which improves the antioxidant enzyme CAT activity and reduces lipid peroxidation to decrease oxidative stress, thereby inhibiting cell apoptosis to alleviate liver damage. These findings demonstrate that CUR/CDP has the potential to become a fresh pharmacological agent to reduce liver injury resulting from oxidative damage.

## CONFLICT OF INTEREST

We declared that there was no conflict of interest.

## AUTHOR CONTRIBUTION

**Jianping Chen:** Conceptualization (lead); Formal analysis (lead); Funding acquisition (lead); Project administration (lead); Writing – original draft (lead); Writing – review & editing (lead). **Jiarui Li:** Data curation (equal); Formal analysis (equal); Investigation (equal); Methodology (equal). **Tugui Fan:** Data curation (equal); Formal analysis (equal); Investigation (equal); Methodology (equal); Validation (equal). **Saiyi Zhong:** Resources (supporting); Supervision (supporting). **Xiaoming Qin:** Resources (supporting); Supervision (supporting). **Rui Li:** Resources (equal); Software (equal). **Jialong Gao:** Resources (supporting); Software (supporting). **Yuanwei Liang:** Funding acquisition (supporting); Project administration (supporting); Resources (supporting); Writing – review & editing (supporting).

## Data Availability

The data are available from the author upon reasonable request.
